# Evaluating Pressure Ulcer Risk With PURPOSE T in Acute Care—Inter‐Rater Reliability Between Registered Nurses and Assistant Nurses

**DOI:** 10.1111/iwj.70946

**Published:** 2026-05-14

**Authors:** Ulrika Källman, Kerstin Ulin, Lisa Hultin

**Affiliations:** ^1^ Department of Research, Education and Innovation Södra Älvsborg Hospital Borås Region Västra Götaland Sweden; ^2^ Institute of Health and Care Sciences, Sahlgrenska Academy, University of Gothenburg Gothenburg Sweden; ^3^ Sahlgrenska University Hospital Gothenburg Region Västra Götaland Sweden; ^4^ Department of Public Health and Caring Sciences Uppsala University Uppsala Sweden; ^5^ Uppsala University Hospital Uppsala Sweden

**Keywords:** nurses, pressure ulcer, primary prevention, reproducibility of results, risk assessment

## Abstract

This study evaluated the inter‐rater reliability and usability of the Swedish version of the Pressure Ulcer Risk Primary or Secondary Evaluation Tool (PURPOSE T) when used by both registered nurses and assistant nurses. Although previous validation studies involved only registered nurses, assistant nurses often perform these assessments in practice. The study was conducted in three acute care hospitals in Sweden between October 2022 and September 2023, involving 117 patients. In pairs, eight registered nurses and eight assistant nurses independently assessed patients using PURPOSE T in paper format. The percentage agreement between the registered nurses and assistant nurses was 86.6% (*n* = 97/112). The corresponding simple kappa statistic was 0.69 (95% CI 0.55–0.84); the PABAK kappa statistic was 0.73 (95% CI 0.61–0.86). Both registered nurses and assistant nurses found the tool generally easy to use, though some items in Step 2 were reported as challenging. While the study indicated good inter‐rater reliability and PURPOSE T was perceived as user‐friendly by both groups, education and training should be prioritised when implementing the instrument in routine care to optimise its use. A digital application may provide more effective facilitation and guidance during the assessment compared with a paper‐based version.

## Key Points

- What is the inter‐rater reliability of the Swedish version of PURPOSE T when used by registered nurses (RNs) and assistant nurses (ANs) in acute care hospitals, and how do they describe the instrument’s usability in clinical nursing practice?
- Why is this important? RNs have the primary responsibility for assessing patients' risk of pressure ulcers. However, they often rely on the expertise of ANs and frequently delegate pressure ulcer prevention measures to them. In addition, ANs usually have more frequent contact with patients than RNs, particularly when performing skin inspections.
- In this study, RNs and ANs assessed patients' risk in pairs, using the Swedish version of PURPOSE T, without consulting each other. The analysis showed that the instrument demonstrated good inter‐rater reliability in clinical practice when used by both RNs and ANs. It was also perceived as user‐friendly although both professional groups reported difficulties with some items.
- Based on these findings, education and training should be prioritised when implementing the instrument in routine care to optimise its use. Ideally, RNs and ANs should perform the risk assessment together at the bedside.

## 1 Introduction

Pressure ulcers (PUs) continue to represent a substantial global healthcare challenge, with prevalence rates in acute care settings reported to range between 11.8% and 23.8%, and incidence rates ranging from 7.6% to 25.9%, depending on the population studied [1, 2]. PUs negatively impact patients' quality of life [3, 4] and impose a considerable financial burden on healthcare systems [5, 6, 7]. Given the resource‐intensive nature of preventive interventions (e.g., repositioning, specialised mattresses), applying them indiscriminately to all patients may be inappropriate, as it can negatively impact quality of life (e.g., sleep disturbances) and divert nursing resources away from other essential care activities.

International and national guidelines recommend risk assessments to identify patients at risk and facilitate timely preventive measures [8]. To support this in clinical practice, PU risk assessment instruments (PU‐RAIs) have been developed and are widely used by nurses in many countries, often preferred over clinical judgment alone [8, 9]. A PU‐RAI that has recently been introduced and validated in a Swedish context, demonstrating a 94.5% agreement and a kappa value of 0.81 (95% CI 0.71–0.90), is the Pressure Ulcer Risk Primary or Secondary Evaluation Tool (PURPOSE T) [10].

The practical advantages of PURPOSE‐T lie in its ability to combine a structured yet adaptable approach, starting with an initial screening to efficiently exclude low‐risk patients, followed by a thorough assessment for those identified as potentially at risk [11]. It emphasises clinical judgement throughout the process, promoting a holistic, individualised approach to care planning rather than relying on a numerical scoring system, as seen in traditional pressure injury risk assessment tools [8, 12].

Previous studies that have validated the PURPOSE T have done so with registered nurses (RNs) as assessors, whose evaluations were compared with those of expert nurses. This reflects the fact that RNs hold the primary responsibility for evaluating patients' pressure ulcer risks. However, RNs often rely on the expertise of assistant nurses (ANs) and commonly assign pressure ulcer prevention duties to them [13, 14]. Research further suggests that ANs typically have more frequent patient contact, particularly in relation to skin inspections [15]. In Sweden, RNs complete a three‐year Bachelor of Science degree in nursing and are registered with the National Board of Health and Welfare [16] whereas ANs complete a three‐year upper secondary school education with a focus on basic nursing care [17]. Given the substantial role of ANs in assessing pressure injury risks, it is essential to examine the agreement between RNs' and ANs' risk assessments when applying the PURPOSE T.

### 1.1 Aim

The purpose of the study was to evaluate the inter‐rater reliability of the PURPOSE T among RNs and ANs working in acute care hospitals and to describe their self‐assessment of the instrument's usability in clinical nursing work.

## 2 Material and Methods

This study has an observational, descriptive and comparative design with a focus on inter‐rater reliability. The study followed the Guidelines for Reporting Reliability and Agreement Studies (GRRAS, see File S1).

### 2.1 Setting and Participants

The study was conducted between October 2022 and September 2023 in three acute care hospitals in Sweden, with bed capacities of approximately 300, 600, and 1600 beds, respectively. RNs and ANs from six wards participated.

#### 2.1.1 Nursing Staff

The inclusion criteria for nursing staff were permanent employment of at least 75% on the unit as an RN or AN, at least 6 months of experience in their professional role, and the ability to work both day and evening shifts. The ward manager selected the staff based on their availability and willingness to participate. The planned number of raters was 12 RNs and 12 ANs but the final sample consisted of eight RNs and eight ANs from the six participating wards.

#### 2.1.2 Patients

Hospital inpatients (≥ 18 years old) were consecutively invited to participate in the study. Patients were excluded if they were receiving end‐of‐life care. Participants were recruited at the participating hospital units by the RNs or ANs working on the ward.

#### 2.1.3 Sample Size Calculation

Previous inter‐rater reliability studies of PURPOSE T have presented rater agreement of 93.4% and 94.5% respectively [10, 11]. A power calculation for the present study was performed based on the assumption of a slightly lower risk‐status agreement of 92% between RN and AN. Given the marginal distributions of 25% non‐risk patients and 75% risk patients, 138 patients were required for the lower bound of the 95% confidence interval for kappa to exceed 0.60.

### 2.2 Data Collection

To reduce the risk of training bias, RNs and ANs received education and training on the use of PURPOSE T through a presentation, vignette‐based case studies, a user manual and guidance from the researcher (LH). The session lasted 60 min, during which participants were encouraged to perform one or two practice assessments prior to evaluating real patients.

The RNs and ANs identified eligible patients within their practice areas and provided a verbal explanation of the study along with an information leaflet. Patients were then invited to provide written informed consent.

Risk assessment with PURPOSE T was conducted by one RN and one AN. To minimise the patient burden, the clinical skin assessment and clarifying questions to the patient were conducted simultaneously by the two raters. However, to reduce the measurement bias, each assessor recorded their findings separately in PURPOSE T and decided the patient's risk status without consultation of each other (independently). The RN or the AN finally filled in demographic data in a short protocol for each patient. This included information about ward speciality, patient's date of birth and gender.

Participating RNs and ANs were, shortly after the completion of data collection, asked to respond to questions through a web‐based survey (Table S1). The questions in the survey were based on a previously conducted qualitative study [18] and focused on how the nurses experienced using the PURPOSE T risk assessment tool compared to their usual methods of risk assessment. The response options were presented in the form of a four‐point Likert scale (ranging from ‘very easy to use’ to ‘very difficult to use’ or ‘strongly agree’ to ‘strongly disagree’), along with the option ‘no opinion/do not know’. Two open‐ended questions asked about the pros and cons of the PURPOSE T in relation to the usual risk assessment tools in use (the Modified Norton scale [19] or the Risk Assessment Pressure Sore scale) [20]. Additionally, background data were collected, such as the employee's age, sex, profession and professional experience in their current role (years).

#### 2.2.1 The Instrument

PURPOSE T includes instructions to support nurses in decision‐making, using a colour‐coded system to weight risk factor items based on epidemiological and scientific evidence, clinical relevance, and their role in the PU causal pathway [11]. The colour scheme classifies risk levels as follows: blue (no problem), yellow (potential risk impact), orange (risk present), and pink (existing PU or scarring).

The three‐step assessment process is structured as follows:

1. Step 1—Screening Assessment: Evaluates mobility (four items: one blue, three yellow) and skin status (four items: one blue, two yellow, one pink). Patients with only blue items are screened out, while those with potential or actual PUs proceed to a full assessment.
2. Step 2—Full Assessment: Analyses independent movement (five items), sensory perception (two items), detailed skin assessment (13 predefined skin sites plus additional sites if needed), PU history (three items), perfusion (three items), nutrition (five items), moisture (three items), and diabetes (two items). Items are categorised as blue, yellow, orange, or pink based on risk levels.
3. Step 3—Final Risk Decision: Based on Step 2 responses, patients are classified as (1) not currently at risk (only blue/yellow items), (2) at risk (any orange item or clinical judgement suggests risk), or (3) PU present or previous PU scarring (any pink item).

This structured approach enables efficient risk stratification and targeted PU prevention strategies. The last author (LH) of this manuscript has translated and validated the instrument in a Swedish context [10, 15, 18] with permission from the originator of PURPOSE T. Permission to use the instrument was also sought for this specific project via email to the originator.

### 2.3 Ethics

Approval to conduct the study was obtained from the Swedish Ethical Review Authority (Reg. no. 2022‐01698‐01 & 2022‐05370‐02) prior to data collection. Patients were provided with both verbal and written information about the study's purpose and the procedures for data collection. Written consent was obtained from the patients. All participants were assured that their participation was voluntary, that they could withdraw at any time without explanation and that confidentiality would be maintained. The study adhered to the Declaration of Helsinki [21], as well as national and local ethical guidelines [22].

### 2.4 Analysis

Descriptive statistics were used to describe the demographic variables. For each assessment, data completeness was evaluated for every component of the PURPOSE T, including the number and percentage of missing item‐level data and the assigned risk categories. Cross‐tabulations and kappa statistics (simple kappa and weighted kappa) with 95% CI were used to assess the inter‐rater reliability of the agreement in the three‐way decision pathway and dichotomous risk status (‘at risk [no pressure ulcer, but at risk and pressure ulcer category 1 or above or scarring]/not at risk’ [no pressure ulcer, not currently at risk]). Prevalence‐adjusted bias‐adjusted kappa (PABAK) statistics were also produced to account for the prevalence of risk status and bias between observers [23].

We used published benchmarks to interpret estimates of the kappa statistics: poor k ≤ 0.20, fair 0.21–0.40, moderate 0.41–0.60, good 0.61–0.80 and very good 0.81–1.00 [24, 25]. SPSS (RRID:SCR_002865) version 28 was used for all descriptive analyses except for the calculation of data completeness, which was performed using Excel Statistics version 2502 (RRID:SCR_017294). The Statistical Analysis System (SAS, RRID:SCR_008567) version 9.4 was used for the kappa statistics.

## 3 Results

### 3.1 Patient Characteristics

In total, 117 patients participated in the study across three different hospitals and six wards (medicine/gerontology, *n* = 3; surgery, *n* = 2; psychiatry ward for the elderly, *n* = 1). The 117 patients recruited were assessed in part or in full using PURPOSE T, resulting in a total of 117 paired assessments. The demographic data of the patients are shown in Table 1. Based on the PURPOSE T assessment conducted by RNs, 22 (18.8%) patients had a category 1 or above PU (Table 1). There were a total of 40 PUs across the 22 patients. PURPOSE T identified 79 (67.5%) patients as ‘at risk’ (Table 1).

**TABLE 1:** Patient characteristics.

Variables	Patients, *N* = 117
Age (years)	
Mean (SD)	75.4 (13.4)
Median (min, max)	78.0 (31, 98)
Missing, *n* (%)	6 (5.1)
Sex, *N* (%)	
Male	48 (41.0)
Female	66 (56.4)
Missing	3 (2.6)
Setting, *N* (%)	
Hospital A	38 (32.5)
Hospital B	10 (8.5)
Hospital C	69 (59.0)
Missing	—
Mobility status, PURPOSE‐T Step 1^a^, *N* (%)	
Walks independently with or without walking aids	29 (24.8)
(a) Needs help from another person to walk	23 (19.7)
(b) Spends all/majority of time in bed/chair	22 (18.8)
(c) Remains in same position for long periods	9 (7.7)
a + b	10 (8.5)
a + c	1 (0.9)
b + c	13 (11.1)
a + b + c	10 (8.5)
missing	—
Pressure ulcer,^a^ *N* (%)	
No pressure ulcer	72 (61.5)
Category 1	7 (6.0)
Category 2 or more serious	14 (12.0)
Pressure ulcer, un‐known category	1 (0.8)
Missing	23 (19.7)
PURPOSE‐T risk categorisation,^a^ *N* (%)	
Secondary prevention/treatment pathway	24 (20.5)
Primary prevention pathway	55 (47.0)
Not currently at‐risk pathway	34 (29.1)
Missing	4 (3.4)

^a^ Assessed by registered nurses.

### 3.2 Nursing Staff Characteristics

The risk assessments were carried out by eight RNs and eight ANs, comprising males (*n* = 2) and females (*n* = 14). The median age was 36 (min 23, max 60) years, and the years of experience working as an RN or AN averaged 8 (min 1, max 40) years among the 13 individuals who responded to the follow‐up questionnaire (Table 2).

**TABLE 2:** Registered and assistant nurse characteristics.

Variable	Registered nurse	Assistant Nurse	In total
	*n* = 6	*n* = 7	*n* = 13
	Missing, *n* = 2	Missing, *n* = 1	Missing, *n* = 3
Age, years			
Median (min, max)	41 (23, 47)	30 (23, 60)	36 (23, 60)
Worked as RN or AN, years			
Median (min, max)	6 (1, 15)	8 (4, 40)	8 (1, 40)
Risk assessment experience; *n* (%)			
Very experienced	1 (16.7)	1 (14.3)	2 (15.4)
Fairly experienced	4 (66.6)	6 (85.7)	10 (76.9)
Fairly inexperienced	1 (16.7)	—	1 (7.7)
Very inexperienced	—	—	—
No opinion	—	—	—
Skin assessment experience; *n* (%)			
Very experienced	1 (16.7)	2 (28.6)	3 (23.1)
Fairly experienced	5 (83.3)	5 (71.4)	10 (76.9)
Fairly inexperienced	—	—	—
Very inexperienced	—	—	—
No opinion	—	—	—
Number of conducted risk assessments with PURPOSE‐T in total (during and before the study): *n* (%)			
6–10	2 (33.3)	4 (57.1)	6 (46.2)
11–15	1 (16.7)	2 (28.6)	3 (23.1)
16–20	—	—	—
21–25	—	—	—
> 26	3 (50.0)	1 (14.3)	4 (30.8)

### 3.3 Compliance and Data Completeness

Data completeness and compliance with the completion guidelines for steps 1–3 in PURPOSE T are presented in Table 3. In Step 1, data completeness for each construct was 100%, except for one case among ANs concerning the item ‘clinical judgement’. In Step 2, data completeness among RNs ranged from 87.1% to 95.0%, while among ANs it ranged from 74.8% to 95.3%. For both groups, the construct with the lowest level of completeness was ‘current detailed skin assessment’ (Table 3).

**TABLE 3:** Data completeness and completion.

Construct	Data completeness				Completion of PURPOSE‐T according to guidance		
	Number of items requiring completion	RN assessment	AN assessment	Denominator (i.e., number of items expected to have been completed)	Completion of PURPOSE‐T according to guidance	RN assessment	AN assessment
**Step 1 Screening**							
Mobility	1 of 4	100% (117/117)	100% (117/117)	All patients, as all were required to complete Step 1 mobility	Completed	117 (100%)	117 (100%)
					Non‐completed	—	—
					Total	117 (100%)	117 (100%)
Skin status	1 of 5 if required	100% (29/29)	100% (34/34)	All patients for whom only the blue box was ticked for Step 1 mobility	Appropriate completion (no mobility limitation)	29 (24.8%)	34 (29.1%)
					Appropriate non‐completion (mobility limitation)	66 (56.4%)	48 (41%)
					Inappropriate completion (mobility limitation)	22 (18.8%)	35 (29.9%)
					Total	117 (100%)	117 (100%)
Clinical judgement	1 of 2	100% (24/24)	96% (24/25)	All patients for whom only blue boxes were ticked for Step 1 mobility and skin status	Appropriate completion (no mobility limitation + normal skin)	24 (20.5%)	24 (20.5%)
					Appropriate non‐completion (no mobility limitation + not normal skin)	5 (4.3%)	9 (7.7%)
					Appropriate non‐completion (mobility limitation)	66 (56.4%)	48 (41.0%)
					Inappropriate completion (mobility limitation + not normal skin)	15 (12.8%)	25 (21.4%)
					Inappropriate completion (mobility limitation + normal skin)	7 (6.0%)	10 (8.5%)
					Not completed although no mobility limitation and normal skin	0 (0.0%)	1 (0.9%)
					Total	117 (100%)	117 (100%)
**Progression to Step 2**		101	107		Appropriate progression to Step 2	94 (80.3%)	92 (78.6%)
					Appropriate non‐progression to Step 2	16 (13.7%)	10 (8.5%)
					Inappropriate progression to Step 2	4 (3.4%)	8 (6.8%)
					Step 2 completed but Step 1 not completed	3 (2.6%)	7 (6.0%)
					Total	117 (100%)	117 (100%)
**Step 2 Full assessment**							
Step 1 Mobility	1 of 4	100% (101/101)	100% (107/107)	All patients who progressed to Step 2, as all were required to complete Step 1 mobility			
Step 1 Skin status	1 of 4 if required	100% (5/5)	100% (9/9)	All patients for whom only the blue box was ticked in Step 1 mobility and had vulnerable skin who progressed to Step 2			
Step 1 Clinical judgement	1 of 2 if required	100% (8/8)	93.3% (14/15)	All patients for whom only the blue box was ticked in Step 1 mobility and skin status who progressed to Step 2			
Analysis of independent movement	1 of 5	90.1% (91/101)	87.8% (94/107)	All patients who progressed to Step 2			
Sensory perception and response	1 of 2	91/101	86.9% (93/107)	All patients who progressed to Step 2			
Moisture	1 of 3	92.1% (93/101)	83.2% (89/107)	All patients who progressed to Step 2			
Diabetes	1 of 2	93.1% (94/101)	84.1% (90/107)	All patients who progressed to Step 2			
Perfusion	At least 1	93.1% (94/101)	91.6% (98/107)	All patients who progressed to Step 2			
Nutrition	At least 1	94.1% (95/101)	95.3% (102/107)	All patients who progressed to Step 2			
Medical device	1 of 2	94.1% (95/101)	91.6% (98/107)	All patients who prog—			
Current detailed skin assessment	13	87.1% (1144/1313)	74.8% (1040/1391)	13 (number of main skin sites) × number of patients who progressed to Step 2			
Previous PU history	1 of 2	95.0% (96/101)	90.7% (97/107)	All patients who progressed to Step 2			
Previous PU details	At least 1	90.9% (10/11)	93.3% (14/15)	All patients reported to have a PU history			
Decision pathway allocated		96.0% (97/101)	98.1% (105/107)	All patients who progressed to Step 2			
**Assessment decision**							
Pathway allocated at Step 1 or Step 3					Appropriate pathway	114 (97.4%)	110 (94.0%)
					Inappropriate pathway	2 (1.7%)	7 (6.0%)
					No pathway selected	1 (0.9%)	0 (0%)

*Note:* Green color means correct assessment flow and orange wrong assessment flow.

Progression/non‐progression to Step 2 was completed in line with the recommended assessment flow for 94.0% (*n* = 110/117) of patients assessed by an RN. For ANs, the corresponding figure was 87.1% (*n* = 102/117) (Table 3). All patients were assigned a Step 1 or Step 3 decision pathway by the ANs, and only one patient was not assigned a pathway by the RNs (Table 3).

### 3.4 Inter‐Rater Reliability

There was agreement in the three‐way decision pathway between RNs and ANs for 82.4% (*n* = 92/112) of paired assessments (Table 4). The corresponding simple kappa statistic was 0.73 (95% CI 0.61–0.83), and the weighted kappa statistic was 0.75 (95% CI 0.65–0.86), indicating good to very good inter‐rater reliability.

**TABLE 4:** Cross tabulation of PURPOSE T decision pathway for registered nurses and assistant nurses.

Registered nurse	Assistant nurse			Total
	Existing PU or scarring from previous PU	No PU, but at risk	No PU, not currently at risk	
Existing PU or scarring from previous PU (%)	23 (20.5)	0	1 (0.9)	24 (21.4)
No PU, but at risk (%)	5 (4.5)	41 (36.6)	8 (7.1)	54 (48.2)
No PU, not currently at risk (%)	1	5 (4.5)	28 (25.0)	34 (30.4)
Total (%)	29 (25.9)	46 (41.1)	37 (33.0)	112 (100)

	At risk		Not at risk	Total
At risk (%)	69 (61.6)		9 (8.0)	78 (69.6)
Not at risk (%)	6 (5.4)		28 (25.0)	34 (30.4)
Total (%)	75 (67.0)		37 (33.0)	112 (100)

*Note:* The gray shaded cells indicates the number of assessments registered nurses and assistant nurses assessed equally.

When classified dichotomously as ‘at risk’ and ‘not at risk’, there was agreement between the RN and AN for 86.6% (*n* = 97/112) of paired assessments (Table 4). The corresponding simple kappa statistic was 0.69 (95% CI 0.55–0.84), and the PABAK kappa statistic was 0.73 (95% CI 0.61–0.86). The simple kappa statistic (CI) indicated moderate to very good inter‐rater reliability, while the PABAK kappa statistic (CI) indicated good to very good reliability.

Table 5 describes the level of agreement between RNs and ANs for specific risk factor items. The lowest level of agreement was for perfusion status (74.7%, *n* = 65/87), and the highest level was for diabetic status (100%, *n* = 79/79).

**TABLE 5:** Levels of agreement between registered nurses and assistant nurses for specific risk factor items.

	Registered nurses vs. assistant nurses	
	*n*=	%
Step 1: Mobility (agreement on presence/absence of problem)	111/117	94.9
Skin status [steps 1 and 2 combined]	90/112	80.4
Step 1: Clinical judgement	22/24	91.7
Analysis of independent movement (agreement on presence/absence of problem)	76/89	88.8
Sensory perception	64/72	88.9
Moisture status (agreement on presence/absence of problem)	67/82	81.7
Diabetes status	79/79	100.0
Perfusions status (agreement on presence/absence of problem)	65/87	74.7
Nutrition (problem vs. no problem)	76/92	82.6
Medical devices	71/90	78.9

### 3.5 Staff Experiences Using PURPOSE T

In total, 13 of 16 staff participants completed the follow‐up questionnaire: six RNs and seven ANs. The participating staff highlighted both positive aspects and challenges of using PURPOSE‐T in practice (see Supporting Information: Appendix). All RNs (*n* = 6) and most ANs (*n* = 6) stated that PURPOSE‐T was generally easy to use. However, some items in Step 2 were found to be more difficult to complete than others. The most challenging items, in order, were previous PU history, perfusion, moisture, and assessment of independent movement (see Table S1).

## 4 Discussion

RNs and ANs assessed 117 patients in this study to evaluate the inter‐rater agreement of PURPOSE T in a Swedish context. The inter‐rater reliability between these two nurse groups, as determined by the simple kappa statistic, was 0.69 for the dichotomous assessment decision (risk/no risk) and the proportion of agreement was 86.6%. Unfortunately, the study was terminated earlier than planned due to the organisation's difficulties in allocating time for data collection. Consequently, we were unable to include the full number of patients specified by the power analysis, which increases the uncertainty of these results. For instance, the simple kappa confidence interval for the dichotomous assessment was very wide (0.55–0.84), indicating RNs and ANs agreement when using PURPOSE T could be ‘moderate’ up to ‘very high’. However, when adjusted with the PABAK‐analysis the lower bound of the confidence interval were above 0.60 indicating ‘good’ to ‘very good’ agreement. Similar with the kappa statistics for the three‐way pathway. These letter results are in line with psychometric properties evaluated in earlier studies of the PURPOSE T, both from Sweden and the UK [10, 11].

The agreement between RNs and ANs on individual items ranged from moderate to high. Similar to the study by Colman et al. [11], participants exhibited the lowest agreement regarding perfusion and the highest agreement concerning the incidence of diabetes. Notably, in the present study, there was a significant difference in skin assessment between RNs and ANs, with agreement lacking in 20% of cases. It is particularly noteworthy that RNs and ANs had differing perceptions in seven cases regarding whether patients had a PU (or a history of one) or not. This indicates that skin assessment is challenging, as stated by others [26]. At the same time, skin status is a key predictor of PU development [27]. Given that considerable emphasis is placed on the results of the skin assessment in PURPOSE T, both in steps 1 and 2, it is crucial that this assessment is as accurate as possible. It was beyond the scope of this study to determine who was right and wrong in the assessment. However, the results suggest that conducting the assessment collaboratively between RNs and ANs could be beneficial.

A more detailed examination of PURPOSE T completion showed that data completeness exceeded 90% for most constructs, indicating that the instrument is generally stable and feasible for clinical use. Nevertheless, higher levels of missing information were observed regarding specific items. Among RNs, incomplete documentation was most commonly noted for the skin assessment item, whereas among ANs, missing data was more frequent for sensory perception, moisture, diabetes, and medical devices. These findings are consistent with previous evaluations of PURPOSE T [10, 11]. One possible explanation of why some item information was lacking is that these risk factors are not included in the PU RAI routinely used in clinical practice and these items may therefore be less familiar to the participating RNs and ANs. On the other hand, when responding to the questionnaire, the nursing staff reported difficulties with other items, including PU history, perfusion, and assessment of independent movement. Overall, the results suggest that a single 60‐min training session is unlikely to be sufficient for either RNs or ANs when implementing PURPOSE T in routine care. More extensive and possibly staged education and training may be required to support adequate understanding of the instrument's constructs and to enable accurate and consistent categorisation of findings. This need may be particularly pronounced given that PURPOSE T is a relatively new assessment tool and includes risk factors that nursing staff may not be familiar with when performing assessments. In this study, PURPOSE T was used in paper format, while most healthcare providers today rely on electronic health records. In addition to education, an electronic version of PURPOSE T could enhance accessibility and better guide staff in its completion.

International PU guidelines recommend incorporating clinical assessment into the risk assessment, as it is considered an essential part of accurate and holistic risk evaluation [8]. Even though the RNs and ANs had not filled in all the risk factors in Step 2, almost all of them performed the final risk decision in Step 3. This indicates that they conducted a clinical assessment based on the patient's condition and the information they either had access to or felt confident in evaluating. Both RNs and ANs emphasised that the results aligned with their perception of the patient's risk of developing PUs, although some items in the assessment were challenging to complete. If a numerical PU‐RAI had been used, the outcome would have been inaccurate if certain risk factors were left unfilled. The construct of PURPOSE T allows assessors to reflect, analyse, and make their own conclusions without conflicting with the RAI result [18].

A central issue in the clinical implementation of PURPOSE‐T concerns who should conduct the risk assessment: the RNs, with a higher level of education and nursing responsibility, or the ANs, who are usually closest to the patient. The findings of this study, together with previous research, suggest that the most appropriate approach may not be one role alone but rather collaboration between the two. Notably, RNs have emphasised in previous studies that it is not possible to perform the PURPOSE T assessment without direct patient contact at the bedside [15, 18]. They emphasise that the instrument not only identifies the risk of PUs but also contributes to an overall picture of the patient's general condition, thereby facilitating consideration of other nursing needs that should be included in the patient's care plan, which, in the best cases, is developed in collaboration with the patient. It could be argued that this workflow consumes unnecessary resources. However, completing the PURPOSE T requires little time [18], and the information obtained is essential for the entire team, including the patient.

### 4.1 Clinical Implications

The study identified discrepancies between RNs and ANs in their skin assessments and in their perceptions of whether patients had a pressure ulcer. Because accurate skin assessment is inherently complex and central to the PURPOSE‐T framework, implementation efforts should include focused training to ensure that both RNs and ANs have the skills required to carry out systematic, high‐quality assessments.

Conducting the risk assessment together at the bedside can also strengthen assessment quality and reduce uncertainty about skin status and potential risk factors. Additionally, a digital application could help guide the process by ensuring all fields are completed and by supporting clinicians through structured decision prompts.

### 4.2 Evaluation of the Study

The research group followed transparent processes throughout data collection and analysis to enhance validity and reliability. This included, for example, the group's involvement in the development of training materials and piloting procedures, reflexive discussions of the data collected, and the use of agreed‐upon analytical procedures.

Several potential sources of bias should be considered when interpreting the findings. First, selection bias may have occurred because the proportion of participating RNs and ANs was relatively low, potentially limiting the generalizability of the results. However, the inclusion of three hospitals and multiple wards strengthens the diversity and contextual relevance of the data.

Second, the total number of PURPOSE‐T assessments performed by the raters (both before and during the study) varied considerably. Although all RNs and ANs received structured training prior to data collection, this variation may still have influenced the results. Moreover, as noted earlier, the 60‐min training session may have been too brief to ensure optimal understanding and consistent application of the instrument.

Third, the possibility of measurement bias cannot be excluded because the RN and AN conducted part of the assessment together. Despite receiving clear instructions not to communicate and to complete their PURPOSE‐T templates independently, some unintentional influence may still have occurred.

Finally, an important limitation is the absence of a true gold standard. In this study, the ratings of registered nurses were used pragmatically as a reference point, given their higher level of education. While this facilitated comparison between the two professional groups, it does not provide an absolute benchmark of accuracy and may therefore introduce bias.

Despite these limitations, the measures taken to reduce bias and the use of established statistical methods provide confidence in the robustness of our findings regarding the inter‐rater reliability and usability of PURPOSE T in clinical practice.

## 5 Conclusion

The results of this study indicate that the Swedish version of PURPOSE T demonstrates good inter‐rater reliability in assessing patients' risk in clinical practice when used by both RNs and ANs. However, the findings should be interpreted with caution as the analysis may be underpowered due to the limited sample size.

Although PURPOSE T appeared to be a user‐friendly instrument from both RNs' and ANs' point of view, education and training should be prioritised when implementing the instrument in routine care to optimise its use. Ideally, RNs and ANs should perform the risk assessment together at the bedside. A digital application may provide more effective facilitation and guidance during the assessment compared with a paper‐based version. Further research on patient involvement in the risk assessment process is warranted.

## Funding

This study was supported by the Local Research and Development Council Södra Älvsborg (VGFOUSA‐977213, VGFOUSA‐964441, VGFOUSA‐990061).

## Ethics Statement

Ethics approval was obtained from the Swedish Ethical Review Authority (Reg. no. 2022–01698‐01 & Reg. no. 2022–05370‐02). Patients were provided with both verbal and written information about the study's purpose and the procedures for data collection. Written consent was obtained from the patients. All participants were assured that their participation was voluntary, that they could withdraw at any time without explanation, and that confidentiality would be maintained.

## Conflicts of Interest

The authors declare no conflicts of interest.

## Supporting information


**Table S1:** Questionnaire to the participating staff.
**Table S2:** RNs' and ANs' opinions on whether some parts of the PURPOSE T were difficult to assess and, if so, which parts. Participants could select one or more options.
**Table S3:** RNs' and ANs' agreement with the statement that the results of PURPOSE‐T are consistent with their perception of the patient's risk of pressure ulcers.

**Data S1:** GRRAS checklist for reporting of studies of reliability and agreement.

## Data Availability

The data that support the findings of this study are available from the corresponding author upon reasonable request.
