# Supplementary material for: Evaluating Pressure Ulcer Risk With PURPOSE T in Acute Care—Inter‐Rater Reliability Between Registered Nurses and Assistant Nurses
Source: Int Wound J. 2026 May 14;23(5):e70946. doi: 10.1111/iwj.70946 (PMC13175763; doi:10.1111/iwj.70946)
Supplement: Supplementary file 1 — Table S1: Questionnaire to the participating staff. Table S2: RNs' and ANs' opinions on whether some parts of the PURPOSE T were difficult to assess and, if so, which parts. Participants could select one or more options. Table S3: RNs' and ANs' agreement with the statement that the results of PURPOSE‐T are consistent with their perception of the patient's risk of pressure ulcers. [file IWJ-23-e70946-s001.docx]

# Supplementary material

Supplement to: Källman U, Ulin K and Hultin L. *Evaluating pressure ulcer risk with PURPOSE T in acute care – inter-rater reliability between registered nurses and assistant nurses*

This appendix has been provided by the authors to give readers additional information about the work.

## Questions to participating staff

The staff completed the survey online (Microsoft Forms) after data collection on the ward was completed.

| Table S1. Questionnaire to the participating staff | |
| --- | --- |
| **Variable/Question** | **Answer option** |
| Gender | Female/Male/Other |
| Age | Year |
| Profession | Registered Nurse/Assistant Nurse |
| Professional experience in current role | Year |
| How do you rate your experience in assessing patients' risk for pressure ulcers? | Very experienced/Fairly experienced/Fairly inexperienced/Very inexperienced/No opinion |
| How do you rate your experience of skin assessment of patients for pressure ulcers? | Very experienced / Fairly experienced / Fairly inexperienced/Very inexperienced/No opinion |
| How many risk assessments have you done with PURPOSE T during the study and before the study? | ≤ 5; 6–10; 11–15; 16–20; 21–25; ≥ 25 |
| What is your opinion on using PURPOSE T? | Very easy to use/Easy to use/Difficult to use/Very difficult to use/No opinion |
| Can you give an example of something that hasn't been good about using PURPOSE T? | Free text |
| Can you give an example of something that has been good about using PURPOSE T? | Free text |
| Of the risk assessments you have done with PURPOSE-T, do you think the results are consistent with your perception of the patient's risk of pressure ulcers? | Strongly agree/Agree/Disagree/Strongly disagree/No opinion |
| Was any part of PURPOSE T difficult to assess? | No/Yes (if yes, tick which item(s) in question below) |
| Which items of PURPOSE T were difficult to assess? Please tick one or more. | All items in PURPOSE T |
| How long do you estimate it will take to conduct a risk assessment with PURPOSE T? | <5; 5–10; 10–15; >15 |
| Which risk assessment tool would you like to use in the future? | Continuing to use what we use today (Modified Norton or RAPS)/ PURPOSE T / Do not know |

## Supplementary tables

Table S2. RNs’ and ANs’ opinions on whether some parts of the PURPOSE T were difficult to assess and, if so, which parts. Participants could select one or more options.

| **Variable** | | **RN, n = 6** | **NA, n = 7** | **Total, n = 13** |
| --- | --- | --- | --- | --- |
|  | | **Missing, n = 2** | **Missing, n = 1** | **Missing, n = 3** |
| Experienced difficulties? | | 1 | 2 | 3 |
| Experienced some difficulties | | 5 | 5 | 10 |
| Risk factors difficult to assess; | |  |  |  |
| Step 1 | Mobility | - | - | - |
|  |  |  |  |  |
|  | Skin status | 1 | - | 1 |
|  | Clinical judgement | - | 2 | 2 |
| Step 2 | Analysis of independent movement | 1 | 3 | 4 |
|  | Sensory perception and response | 1 | 1 | 2 |
|  | Moisture | 1 | 3 | 4 |
|  | Diabetes |  | 1 | 1 |
|  | Perfusion | 3 | 2 | 5 |
|  | Nutrition | 1 | - | 1 |
|  | Medical device | - | - | - |
|  | Current detailed skin assessment | - | - | - |
|  | Previous PU history | 3 | 3 | 6 |
| Step 3 | Final decision | - | 1 | 1 |

| Table S3. RNs’ and ANs’ agreement with the statement that the results of PURPOSE-T are consistent with their perception of the patient's risk of pressure ulcers. | | | |
| --- | --- | --- | --- |
|  | **RN**  **n = 6** | **AN**  **n = 7** | **Total**  **n = 13** |
| Strongly agree, n = (%) | 4 (66.7) | 7 (100) | 11 (84.6) |
| Agree, n = (%) | 2 (33.3) | 0 (0.0) | 2 (15.4) |
| Disagree | - | - | - |
| Strongly disagree | - | - | - |
| No opinion | - | - | - |

**Pros and cons described by participating staff about PURPOSE T**

The following comments were written by RNs and ANs about the disadvantages of PURPOSE T:

RNs:

- “Sometimes it's difficult to assess what should be filled in”
- “It's harder to assess when only yellow markings are checked”
- “There are many ways to interpret the questions, which leads to varying assessments”
- “Pain is missing as a risk factor”

ANs:

- “Takes a little more time [compared to the instrument they use today]”
- “The question about normal skin is very complex. In Cosmic [the patient record system], it seems to be related to whether or not the patient has a pressure ulcer, but many patients do not have normal skin”
- “Also Step 2, where you are supposed to fill in, for example, high BMI, low BMI, cardiovascular diseases, etc.; pain is missing”

The following comments were written by RNs and ANs about the advantages of PURPOSE T:

RNs

- “Easy to use and easy to understand”
- “Easy to follow, with additional assessment based on diabetes, which is not included in RAPS [the ordinary risk assessment tool]”
- “The background questions are relevant, and it's good that you can assess based on your clinical judgment”
- “Being able to assess whether the patient has a pressure ulcer or not and then create a care plan to implement measures that minimise the risk of pressure ulcers”
- “It helps me reflect on the patient's individual risks”
- “It's a good tool because it ensures that you don't miss either existing pressure ulcers or the risk of developing them – simple, and the arrow system is helpful”

ANs

- “You can really keep full track of the patient because you ask many more questions if there is a risk”
- “It became easier to understand after participating in the study. It's good to go through the different steps”
- “You gain more insight into the patient's skin condition and previous pressure ulcers”
